# Enhanced paramagnetism of mesoscopic graphdiyne by doping with nitrogen

**DOI:** 10.1038/s41598-017-11698-9

**Published:** 2017-09-14

**Authors:** Mingjia Zhang, Xiaoxiong Wang, Huijuan Sun, Ning Wang, Qing Lv, Weiwei Cui, Yunze Long, Changshui Huang

**Affiliations:** 1 Qingdao Institute of Bioenergy and Bioprocess Technology, Chinese Academy of Sciences, Qingdao, 266101 P. R. China; 2 Department of Physics, Qingdao University, Qingdao, 266071 P. R. China

## Abstract

The new two-dimensional graphitic material, graphdiyne, has attracted great interest recently due to the superior intrinsic semiconductor properties. Here we investigate the magnetism of pure graphdiyne material and find it demonstrating a remarkable paramagnetic characteristic, which can be attributed to the appearance of special *sp*-hybridized carbon atoms. On this basis, we further introduce nitrogen with 5.29% N/C ratio into graphdiyne followed by simply annealing in a dopant source and realize a twofold enhancement of saturation moment at 2 K. Associate with the density of states calculation, we investigate the influence of the nitrogen atom doping sites on paramagnetism, and further reveal the important role of doped nitrogen atom on benzene ring in improving local magnetic moment. These results can not only help us deeply understand the intrinsic magnetism of graphdiyne, but also open an efficient way to improve magnetism of graphdiyne by hetero atom doping, like nitrogen doping, which may promote the potential application of graphdiyne in spintronics.

## Introduction

The excellent transport properties of low-dimensional carbon materials such as graphene, carbon nanotube have attracted much attention because of the potential application prospect in future electronic device^1–3^. Especially the combination of extraordinary carrier mobility and long spin diffusion makes these materials promising in spintronics or information storage devices^4^. However, the magnetic properties of carbon materials are still quite weak, for instance the intrinsic paramagnetic magnetic moment of graphene is limited to 0.02 emu/g^5^, which poses a rigorous challenge for carbon-based spintronic devices. Meanwhile, the high conducting graphene has no energy gap, limiting its application in semiconductor industry^6^. Thus it is needed to develop new carbon material system with larger magnetic moment as well as excellent semiconducting characteristics before expending the low-dimensional carbon materials in spintronics. More importantly, the investigation of magnetism on novel carbon system can help us reveal a widely applicable interaction mechanism among lattice, spin and orbit, further guiding the manipulation of magnetic moment with different synthetic process.

Recently, a new two-dimensional (2D) carbon material graphdiyne (GDY), has been successfully prepared by cross-linking reaction^7^ and drawn much attention due to its fascinating properties^8, 9^. As an allotrope of graphene with direct band gap structure near Dirac cone, graphdiyne is confirmed as a semiconductor with a band gap of about 1 eV^9, 10^, and its mobility can be even comparable with graphene^11^. The unique structure and acetylenic bond have made GDY beneficial for carrier transport with the mobility as high as 10^4^~10^5^ cm^2^ V^−1^ s^−1^ ^12^, demonstrating a great application potential in carbon-based electronic devices. Meanwhile, based on the first-principles calculations, the two-dimensional carbon material containing *sp*- and *sp* ^*2*^-hybridized carbon atoms such as graphyne has been found that its magnetic ground state and magnetic moments depend on the edge state and the width of the zigzag-like ribbons^13^. However, the magnetism of GDY has not yet been investigated deeply, which is very important not only to reveal spin polarization state in this 2D carbon material but also to develop graphdiyne-based spintronic devices^14^. More importantly, doping is the practice of adding atoms of impurities to a semiconductor lattice to modify electrical structure and properties. The advantages of graphdiyne for spintronics lie in doping tunable bandgap and conductivity as well as easy-realizable atomic substitution or modification among its special structure^9^. For example, it has been reported that transition metal atoms can be easily introduced into graphdiyne by interacting with acetylene linkage^15^. Thus graphdiyne can be regarded as one promising candidate material for spintronics by element doping. Recently, graphdiyne doped with nitrogen is more desirable for high-powered energy storage device^16^, oxygen reduction reaction^17^ and so on, indicating a wide application prospect with the substitution of nitrogen. On the aspect of magnetism, it has been confirmed in theory that nitrogen (N) doping is an effective method to introduce local magnetic moment^18^. Therefore, we can anticipate nitrogen doping an effective way to further manipulate the magnetism of GDY.

In this study, we for the first time investigate the magnetic characteristics of graphdiyne powder by magnetic moment measurement with temperature changing from 2 K to 300 K. It is found that pure graphdiyne show a typical paramagnetism with a large saturation moment *M*_*s*_ = 0.51 emu/g at 2 K, which arise from the unique carbon atom matrix and *sp*-hybridization of graphdiyne. Besides, by introducing nitrogen into graphdiyne, the paramagnetism is further enhanced by nearly two times, revealing the import role of pyridine nitrogen in local magnetic moment enhancement for 2D carbon materials. These results indicate that graphdiyne can be expected to realize stronger magnetism by the regulation of synthesis, suggesting that graphdiyne could be a very promising spintronic material among the 2D carbon material family.

## Results

**Figure 1:**
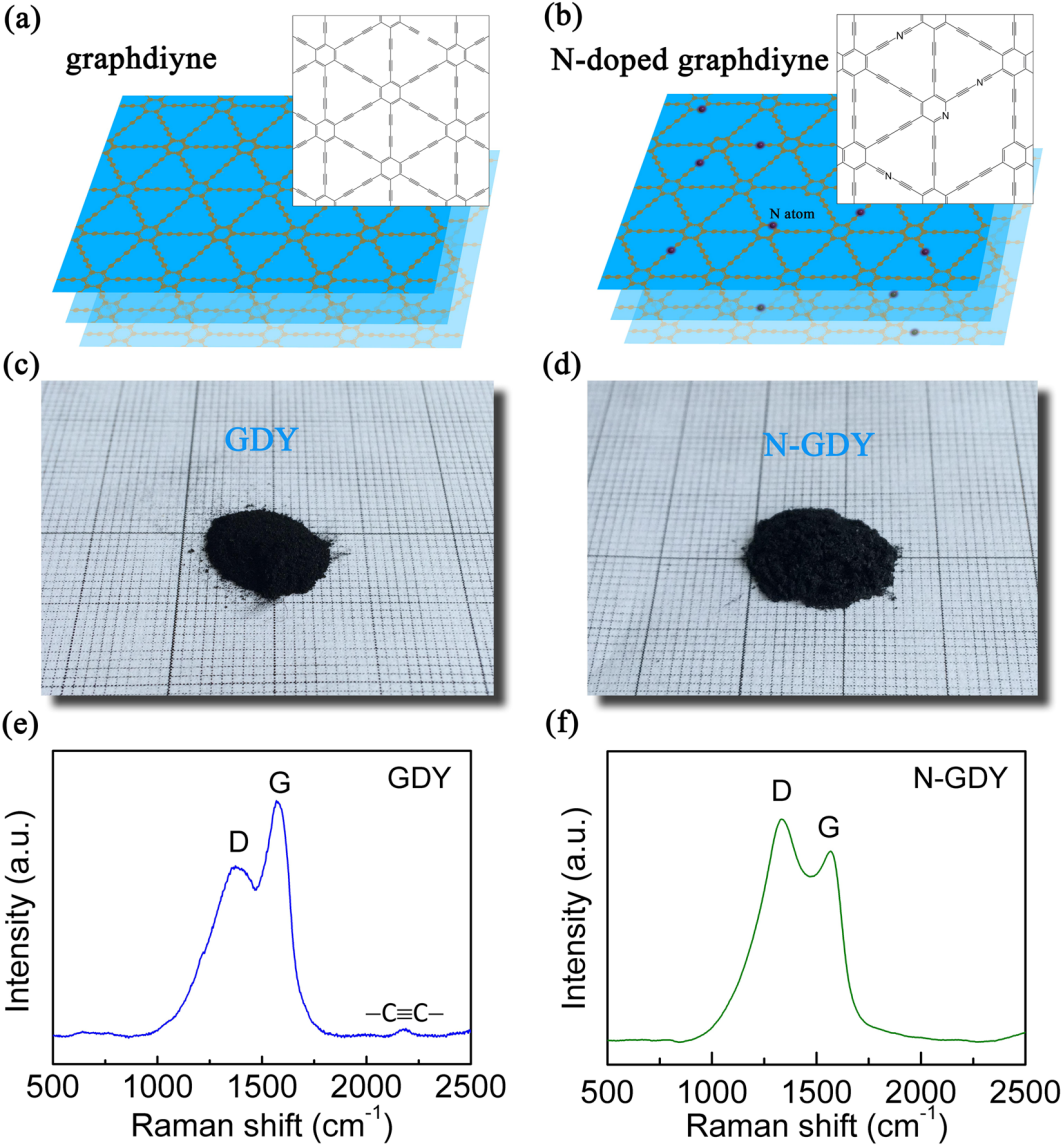
(**a**,**b**) Structure diagrams of GDY and N-GDY. (**c**,**d**) Optical photographs of the GDY powders without and with nitrogen doping respectively. (**e**,**f**) Raman spectrum of pristine and N-doping GDY.

The graphdiyne film was synthesized via a cross-coupling reaction based on the previous report^7^. The structure diagrams of GDY and nitrogen-doped GDY (N-GDY) are sketched in Fig. 1a and b respectively. Figure 1c and d are the optical photographs of the corresponding powder samples, which indicate that the macroscopic feature of our samples shows no obvious change by nitrogen doping. Figure 1e and f display the Raman spectra of the GDY powders without and with N-doping respectively, and it can be seen that both D-band and G-band are obviously distinguished. The G-band at 1577,5 cm^−1^ and 1568.3 cm^−1^ for GDY and N-GDY arises from the E_2g_ stretching vibration mode^7^. The D-band, which is related with structural defects such as edges and disordered carbon atoms, shows a relative enhancement compared with G-band after N-doping. Here we can use the intensity ratio of D-band and G-band (*I*_*D*_/*I*_*G*_) to evaluate the degree of structural deformation. The calculate *I*_*D*_/*I*_*G*_ are 0.71 and 1.16 for GDY and N-GDY respectively, suggesting that the pure GDY is ordered with low content of defects^19^ while N-doping can introduce vacancies and disorder efficiently. Meanwhile, the Raman peak at 2183.2 cm^−1^ for GDY, which corresponds to the vibration of acetylenic linkages (−C≡C−C≡C−)^20^, becomes weaker for N-GDY due to the substitution of N atoms in the triple-bonded carbon atoms.

**Figure 2:**
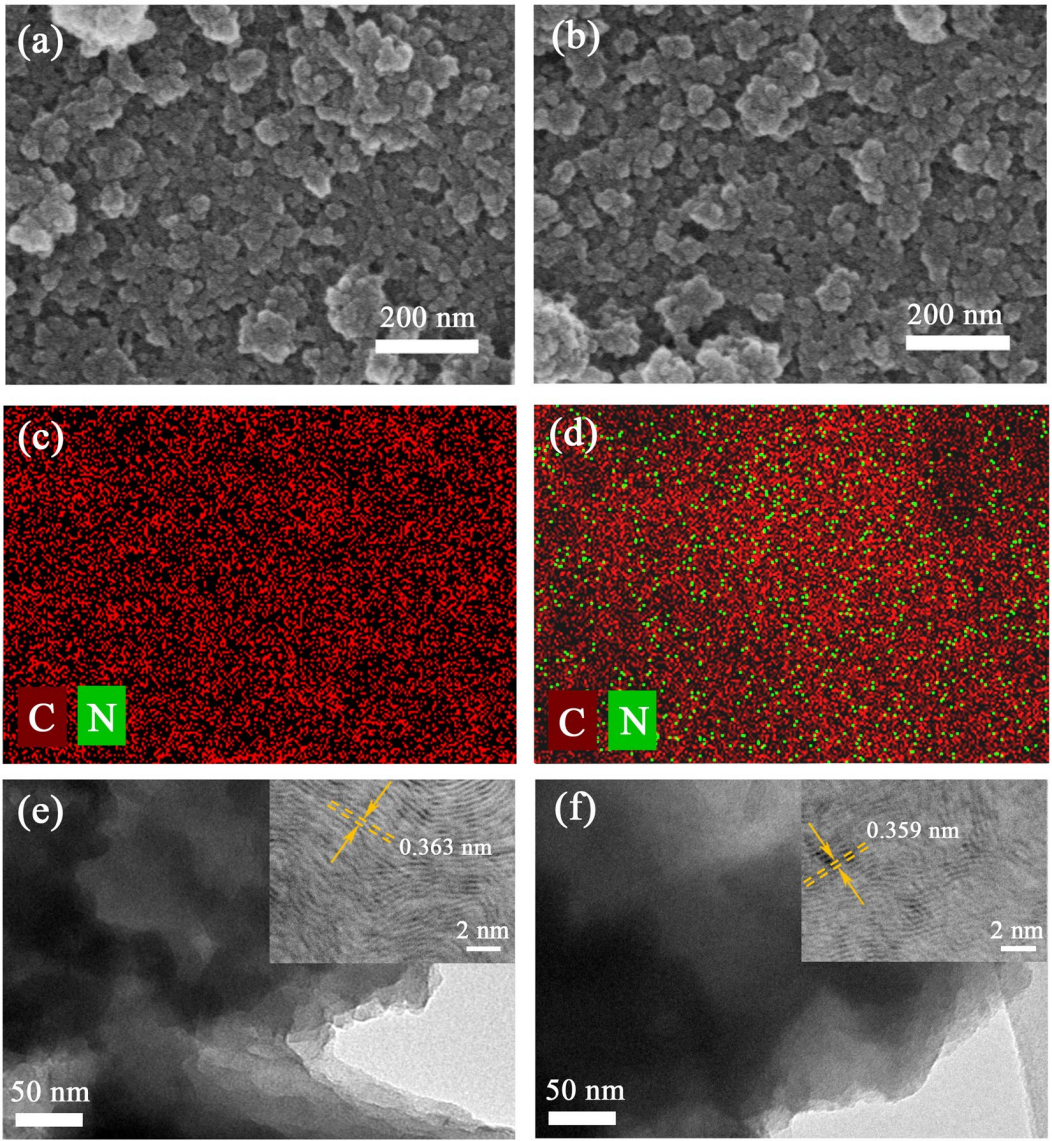
SEM images of (**a**) GDY and (**b**) N-GDY. (**c**) Distribution of carbon atoms on the GDY sheet. (**d**) Combined C/N chemical mapping of the N-GDY sheet. (**e**,**f**) TEM images for GDY and N-GDY respectively. Insets are the corresponding HRTEM images.

Figure 2a and b show the scanning electron microscopy (SEM) images of the raw GDY powder and N-GDY powder respectively. It can be seen that both the morphology demonstrate the agglomeration of nano-size particles and the GDY powder maintains its initial morphology after N-doping. The distribution of nitrogen and carbon within the nano sheet is investigated by scanning electron microscope chemical mapping (Fig. 2c and d), which indicates that the nitrogen atoms are effectively introduced into GDY after NH_3_ treatment as well as the nitrogen groups are distributed rather homogeneously within the carbon matrix (Fig. 2d) for N-GDY. Besides, the transmission electron microscope (TEM) images are also obtained to demonstrate the uniform and continuous microstructure with stacked layers, which is shown in Fig. 2e and f. The inserted high-resolution (HR) TEM images further demonstrate that the layer structure and interlayer spacing have no significant change after N-doping.

**Figure 3:**
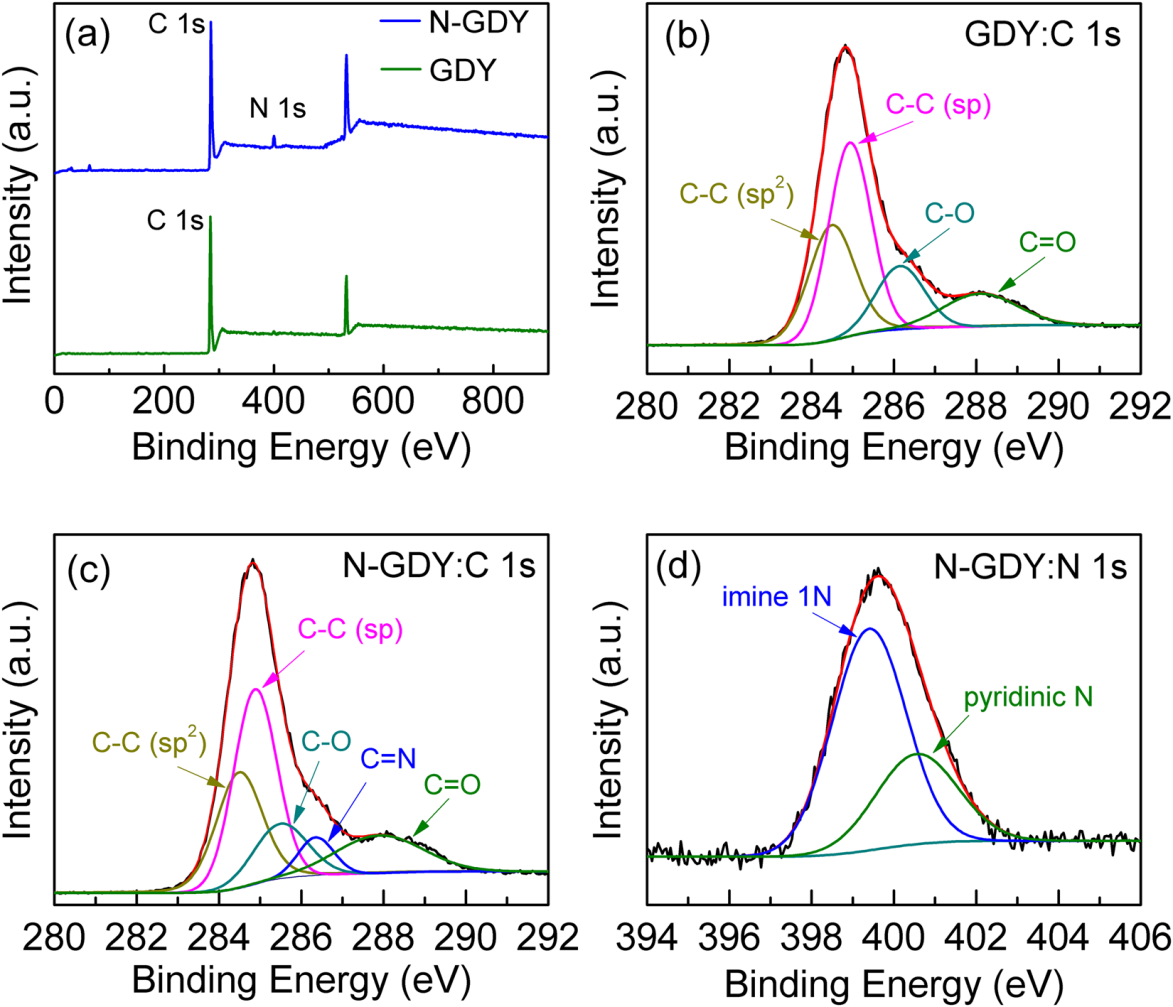
(**a**) XPS spectra of GDY and N-doped GDY. (**b**,**c**) The fine-scanned C 1 s XPS spectra of GDY and N-GDY respectively. (**d**) Fine-scanned N 1 s XPS spectrum of N-GDY.

The overall chemical composition of GDY and N-GDY samples are determined by X-ray photoelectron spectroscopy (XPS) to revel the average contents of carbon and nitrogen, which is shown in Fig. 3a. The C 1 s peak indicates the binding energies for the C 1 s orbital and the appearance of O 1 s peak arises from the absorption of air in the pore of the carbon network in graphdiyne. Especially an obvious N 1 s peak is found at 399.9 eV for N-GDY, and the atomic ratio of N/C is estimated about 5.29, suggesting the efficient introduction of nitrogen in GDY. Figure 3b shows a high resolution C 1 s XPS spectrum of GDY and it can be fitted and divided into four bonds including C-C (sp^2^), C≡C (sp), C-O and C=O^19^. For N-GDY, the C 1 s peak should be assigned to five bonds such as C-C (sp^2^), C≡C (sp), C-O, C=N and C=O^17, 21^, as Fig. 3c shown. Besides, it has been reported that the area ratio of sp/sp^2^ can be used to evaluate the connecting characteristic of benzene rings by diine. For our N-GDY sample, the ratio of sp/sp^2^ is reduced to 1.74 compared with that of GDY, which indicates that the efficient substitution of Nitrogen on sp-carbon reduces some of the C≡C and results in the formation of C=N^17^. To further analyze the nitrogen doping characteristic, the N 1 s peak of N-GDY can be fitted based on Gauss–Lorenz function to reveal the possible doping positions of N atoms^16, 21^. As shown in Fig. 3d, the N 1 s peak shows two components which are assigned to two typical peaks, suggesting that the N atoms can bond with C atoms in the GDY matrix in the form of imine N (N atom substitution on acetylene linkage such as the sp-hybridized carbon atoms) or pyridinic N (N atom substitution on benzene)^17^. In detail, the strong peak at 399.1 eV can be attributed to imine 1 N, and another peak at 400.2 eV is contributed by imine 2 N or pyridinic N^16^, which is shown in Fig. 3d. According to the DFT calculation of XPS N 1 s values, the carbon atoms between two adjacent acetenyl groups can be substituted by one or two nitrogen atoms, which corresponds to the imine 1 N or 2 N doping models^17^. Thus the peak-fit processing results reveal that the possible doping positions of N atoms in carbon matrix of graphdiyne lie in either benzene rings position or acetylenic link position.

**Figure 4:**
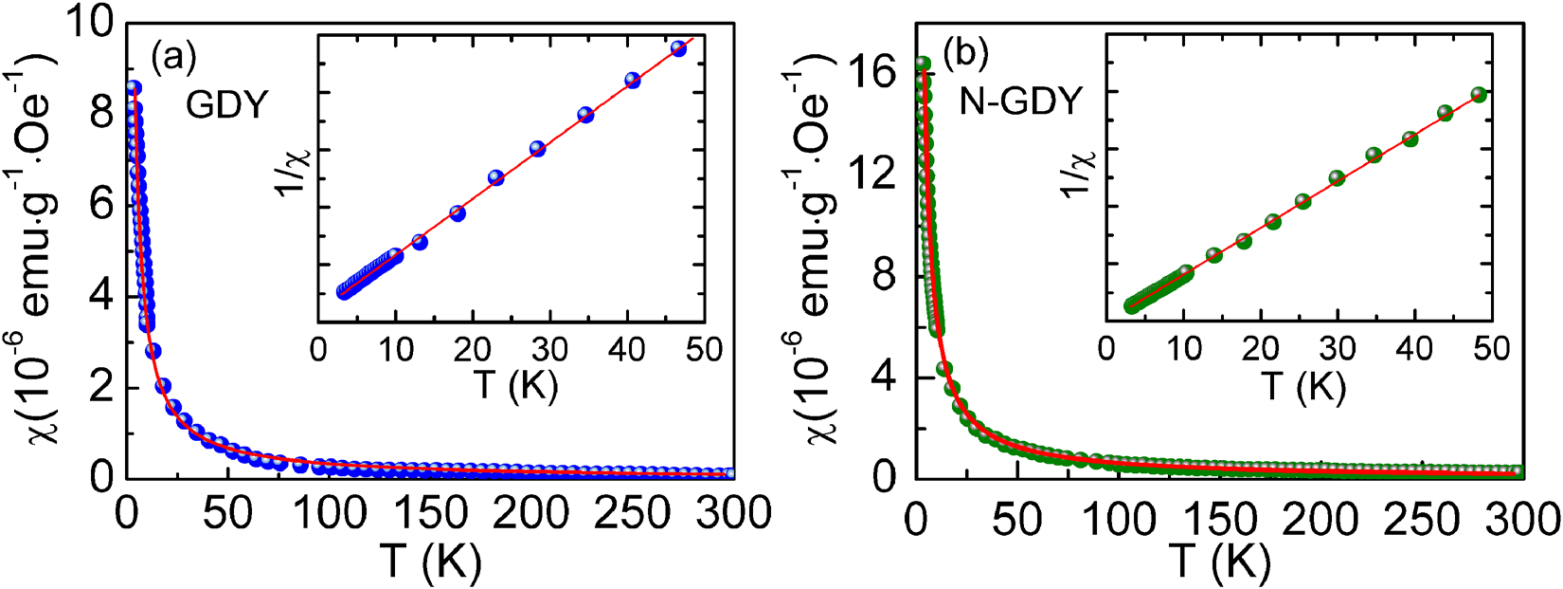
Typical χ-T curves measured from 2 K to 300 K under the applied field H = 5 kOe for (**a**) GDY and (**b**) N-GDY. The solid line if fitted by the Curie law. Insets are the corresponding 1/χ-T curves.

To investigate the intrinsic magnetism of GDY material, we measured its magnetic moment with different magnetic fields and temperatures. Figure 4 shows the temperature dependent magnetic susceptibility *χ*-*T* curves of GDY and N-GDY with temperature ranging from 300 K to 2 K. We can see that both the magnetic susceptibilities increase with decreasing temperature especially at low temperature, and no obvious magnetic transition can be observed, suggesting a typical paramagnetic characteristic for GDY and N-GDY. Here, the temperature dependence of susceptibility χ can be described as the Curie-like paramagnetic behavior by fitting with the Curie law $\chi =NJ(J+1){g}^{2}{\mu }_{B}^{2}/(3{k}_{B}T)$ as the solid line shown. The insets correspond to 1/χ~T curves and the linear relation further confirms that there is no sign of ferromagnetic ordering in our samples.

**Figure 5:**
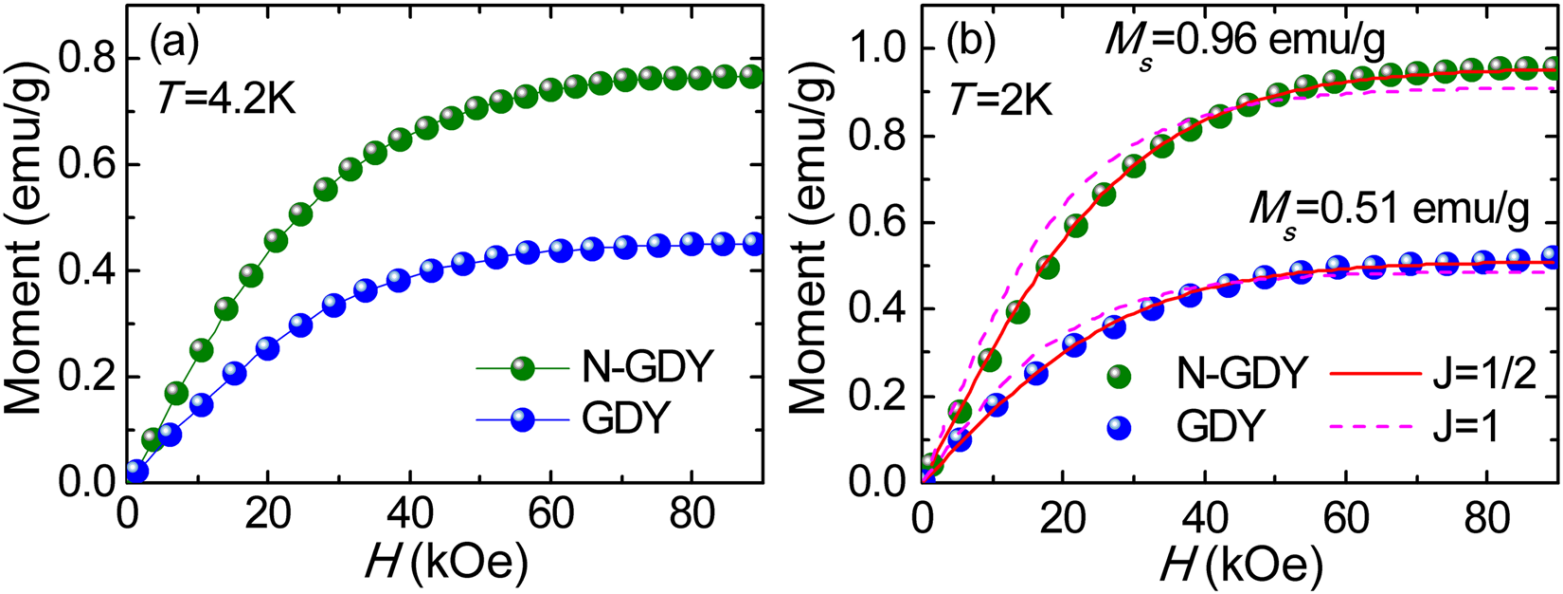
M-H curves measured at (**a**) 4.2 K and (**b**) 2 K for GDY and N-GDY. The solid and dashed lines are fits to the Brillouin function with J = 1/2 and J = 1 respectively.

Further, the typical magnetization curves for GDY and N-GDY are also measured at liquid helium temperature 4.2 K (Fig. 5a), liquid nitrogen temperature 77 K (Fig. S1) and room temperature 300 K (Fig. S2) respectively. At 4.2 K, with the increase of magnetic field *H*, the moment increases rapidly and then trends to steady due to almost all spin arranged along the magnetic field, also confirming the characteristic of paramagnetism. To obtain the saturation magnetization value as well as gain a further insight into the origination of magnetism, we also measured and analyzed the *M-H* curves of GDY and N-GDY respectively at 2 K, which is shown in Fig. 5b. The saturation magnetization of GDY exhibits much larger than that of intrinsic graphene (see Supporting Information, Fig. S3)^5, 22^, suggesting that the GDY material is a more promising candidate for carbon magnetic materials. Meanwhile, it is obvious that the nitrogen doping significantly increases the paramagnetic moment by nearly two times, demonstrating a simple method to regulate the magnetism of graphdiyne. Herein, the *M-H* curves at 2 K can be described by the Brillouin function as fellows^23^:

1 $$M={M}_{s}[\frac{2J+1}{2J}cont(\frac{2J+1}{2J}x)-\frac{1}{2J}cont(\frac{x}{2J})],$$

where *M*_*s*_ is the saturation magnetization, *x* = *gJμ*_*B*_ *H*/*k*_*B*_ *T*, *k*_*B*_ is the Boltzmann constant, *μ*_*B*_ is the Bohr magneton, *J* is the angular momentum quantum number and *g* is the g-factor. As the solid lines shown in Fig. 5b, the experimental data of GDY can be well fitted by Brillouin function for *g* = 2, *J* = 1/2 (solid line) rather than *g* = 2, *J* = 1 (dashed line), which coincides with the contribution of defects such as vacancy or edge on magnetism in theory^24, 25^. Meanwhile, the fitting parameter *J* = 1/2 also suggests that the magnetization GDY arises from the contribution of electronic magnetic moment without any coupling between these spins. Based on the fitting, the calculated *M*_*s*_ values at 2 K for N-GDY and GDY are 0.96 emu/g and 0.51 emu/g respectively, indicating the slight nitrogen doping can significantly improve the intrinsic magnetism of graphdiyne. On the other hand, the magnetic moment value of N-GDY with 5.29% N/C ratio is comparable with that of fluorinated reduced graphene oxide^26^, meanwhile the conductivity of GDY is also increased to 6.25 × 10^−4^ S·m^−1^ from 3.60 × 10^−4^ S·m^−1^ (Fig. S4), suggesting that GDY is easier to realize the control of electromagnetic property by element doping or substitution.

## Discussion

**Figure 6:**
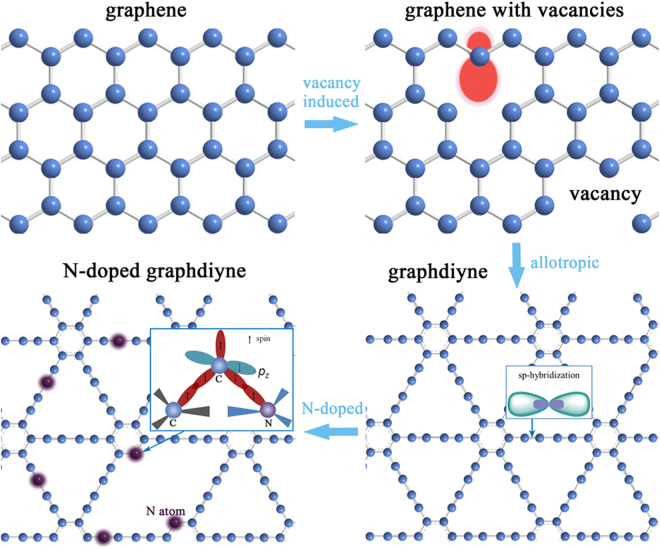
Schematic structure diagrams of graphene and graphdiyne. Insets display the schematic of local orbital interaction.

Based on the unique structure characteristics similar as graphene, we can analyze the origin of paramagnetism in graphdiyne by considering the reported theoretical investigations on magnetism of graphene. The structure and electron orbital for graphene and graphdiyne are depicted schematically in Fig. 6. For graphene, its intrinsic magnetism presents a paramagnetic property with small moment. After introducing vacancy, a magnetic moment in the π band will be induced by creating a sublattice imbalance based on Lieb’s theorem^27^. It has been reported that local magnetic moments may form around each single graphite vacancy and these missing atoms can be considered as a source of carbon magnetism^28, 29^. As for our graphdiyne, it can be regarded as a hybrid system of graphene (sp^2^ hybridization) and carbyne (sp hybridization) with flat carbon network. It has been reported that *sp*-hybridized carbon atoms can contribute local polarized spins and thus introduce an intrinsic magnetic moment based on spin polarized density functional theory calculation^30^. In GDY, the formation of acetylenic bond causes the *sp*-hybridization, which gives rise to the locally enhanced magnetic moment among the whole carbon matrix. On the other hand, the influence of the edge states can’t be ignored. The common edge states in two-dimensional carbon materials such as graphene and graphdiyne are armchair and zigzag. For our graphdiyne, the lamellar stacking characteristic enhances the existing probability of different edge states such as armchair and zigzag, which may be due to inadequate polymerization reaction in the practical process of synthesis. It has been confirmed in theory by first-principles calculation that a magnetic ordering exists at each zigzag edge^13^, thus we can predict that the possible zigzag edge may also contribute to the introduction of local magnetic moment.

As for our graphdiyne with N-doping, the N atoms can exist in different sites as substitutional atoms or adatoms^18^, which are also sketched in Fig. 6. On one hand, substitutional doping of the nitrogen atom has been proven the most favorable and stable among the possible configurations of nitrogen impurities^31^. The possible substitutional sites of nitrogen atoms in graphdiyne are acetylene linkage and benzene ring. Here it should be noted that though the substitution of nitrogen on acetylene linkage will reduce the proportion of *sp*-hybridization carbon, the whole magnetic moment still increases, which indicates the different origins of magnetism between GDY and N-GDY, suggesting the different role of N-doping modes in carbon magnetism. On the other hand, the nitrogen adatoms may migrate to replace carbon atoms to form a stable configuration with C adatoms since the carbon vacancy-adatom pair often appears as one of the most common defects in carbon nanostructure^32, 33^, which may also induce a local magnetic moment^18^. The introduction of nitrogen especially in benzene ring site may result in the interaction between carbon adatoms and N/C atoms^18^. As the inset of Fig. 6 for N-doped graphdiyne shown, two valence electrons form a bond interaction with the N/C atoms. Meanwhile the dangling *sp* ^2^ orbital of adatom C trends to bond with the *π* orbital, thus more electrons can retain in the *p*_*z*_ orbital and be spin-polarized, enhancing the local spin-polarization^18^.

**Figure 7:**
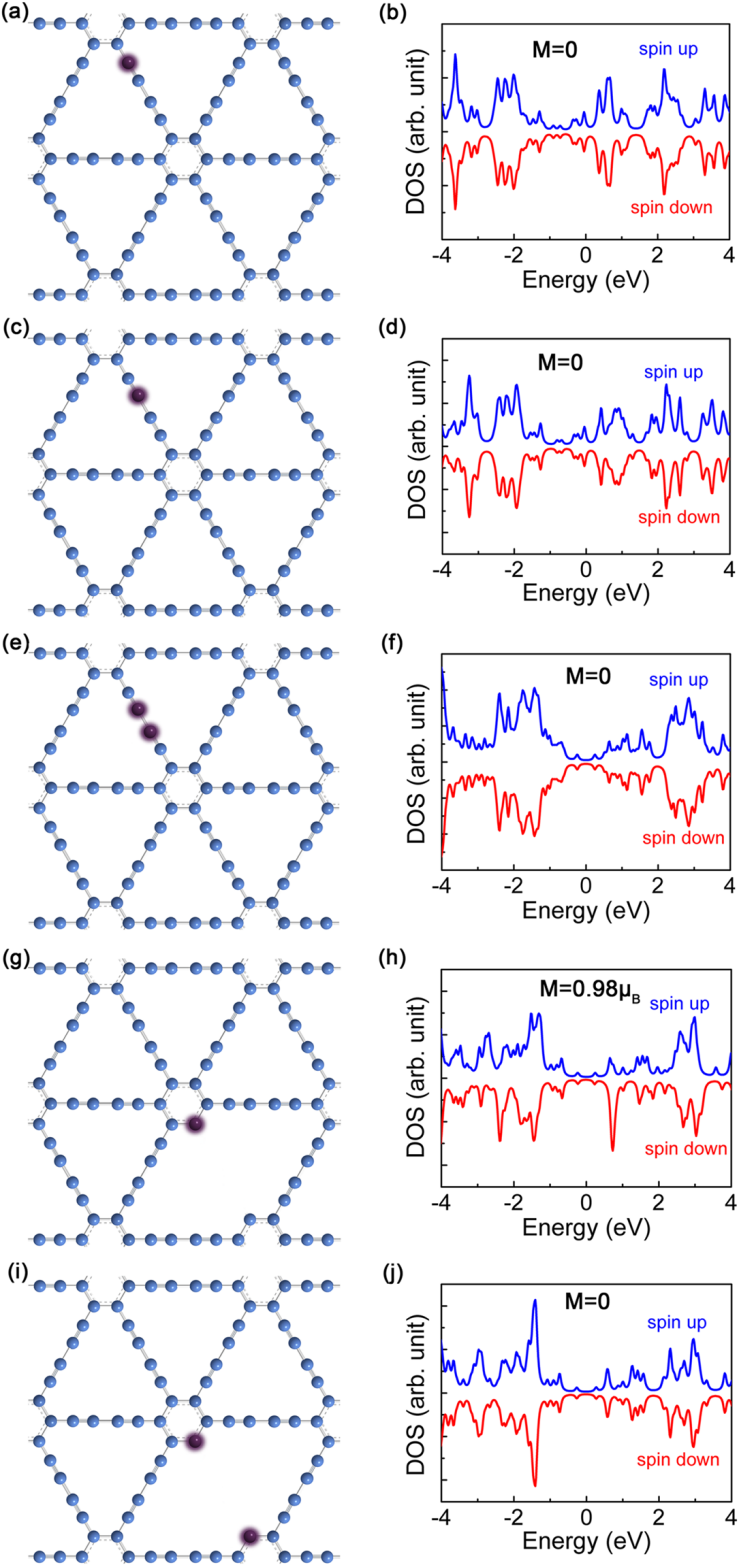
Spin-resolved DOS of N-GDY sheets with doped N atoms on different positions respectively.

To further reveal the influence of nitrogen doping site on local magnetic moment, the electronic structures displayed as density of states (DOS) of N-GDY with substituted nitrogen atoms on acetylene linkage and benzene ring (the simulation models with typical unit cells can be found in Supporting Information, Fig. S5) are calculated by density functional theory formalism with different doping positions of N atoms as Fig. 7 shown respectively. Herein the N-doped GDY models containing five representative unit cells are sketched based on the XPS analysis discussed above. The spin-polarized calculation indicate that an obvious local magnetic moment appears for the asymmetric pyridinic nitrogen substitution (Py-1N) while no magnetic moment can be found for the models with symmetric two pyridinin nitrogen (Py-2N) or nitrogen atom on acetylene linkage, suggesting that the doping mode with pyridine nitrogen is more beneficial to enhance magnetism of graphdiyne. Besides, based on the DFT calculation, we can obtain the local moment of 0.98 μ_B_ for the local structure displayed as Fig. 7g shown. Considering the relative change of measured magnetic moments between N-GDY and raw GDY, it can be concluded that the form of Py-1N may account for a proportion of 13.6% among the possible five kinds of N-doping positions, further revealing the important role of pyridinic nitrogen in improving magnetic properties of chemically modified graphdiyne. However, since the local magnetic moment may not interact with each other to form long-range exchange interactions, none ordered ferromagnetism/ferrimagnetism has been observed. Thus we can expect that the magnetic ordering may be realized in graphdiyne materials by enhancing local spin polarization with further increasing pyridine nitrogen content based on molecular design. These results can not only guide us deeply explore the magnetism of 2D carbon materials but also help us further promote the development of GDY on spintronics devices.

## Conclusion

In conclusion, we prepared high-quality graphdiyne powder samples and investigated their magnetic properties. Structure and morphology characterizations have shown that the GDY is polymerized only with C atoms by *sp* and *sp* ^*2*^ hybridization. Based on the measurement of low-temperature *M-H* curves and temperature dependent magnetic moment, we demonstrate experimentally that GDY is a typical paramagnetic material without obvious magnetic ordering. Compared with other 2D carbon systems, the saturation moment of GDY at 2 K is as high as 0.51 emu/g, which is much larger than the reported intrinsic graphene or graphene oxide. This paramagnetism may be caused by specific *sp* hybridization and carbon atom construction. Furthermore, we also realize an enhanced paramagnetism with the substitution of nitrogen by a simple annealing in ammonia gas, revealing the contribution of nitrogen to local magnetic moment. These results can guide us to further improve the magnetism of GDY by controlling synthesis process, promoting this new 2D-carbon material system to potential applications in organic magnet or spintronic devices.

## Methods

### Synthesis of N-doped graphdiyne

Graphdiyne film on the surface of copper was synthesized via a cross-coupling reaction using the monomer of hexaethynylbenzene based on the previous report^7^. Then the GDY flakes were collected by copper corrosion with hydrochloric acid, washing with acetone and drying in sequence. The N-doped GDY was obtained by annealing GDY flake in the quartz tube at 500 °C for 2 h in NH_3_ atmospheric pressure. Both pure GDY and N-doped GDY samples were ground into powders using an agate mortar respectively. The mass of the GDY powders was accurately determined by High Precision Electronic Balances with a sensitivity of 0.01 mg.

### Characterization of the samples

The X-Ray photoelectron spectrometer (XPS, ULVAC-PHI) was carried out to investigate the bonding environment and chemical component. The structure characteristic of GDY was analyzed by using Raman spectra (NT-MDT NTEGRA Spectra System). Morphological information was measured using scanning electron microscope (Hitachi S-4800 FESEM). Magnetic moment measurements were recorded using a vibrating sample magnetometer (PPMS-VSM, Quantum Design) with temperature changing from 2 K to 300 K.

### The DFT calculation methods

The atomistic calculations are performed based on the spin-polarized DFT using the Vienna ab initio simulation package (VASP)^34–36^. Projector augmented wave (PAW) method is used to describe the electron-nuclear interactions and the exchange correlation energy is described with the Perdew-Burke-Ernzerho (PBE) function^37, 38^. The energy cutoff for the truncation of the plane wave basis is taken to be 500 eV. The geometries are optimized with all forces ≤0.01 eV/Å, and the criterion of convergence for electronic structure is set to be 10^−6^ eV. A DFT optimized lattice constant a = b = 9.29 Å is used here, and the vacuum region is set to be 20 Å to avoid the interlayer interactions. A gamma centered 4 × 4 × 1 k-point mesh is used. Two configurations for the N-doped graphdiyne are considered, with N atom substituted in the acetylene linkage and benzene ring, respectively.

## Electronic supplementary material

revised supporting information
