# Supplementary material for: Enhanced paramagnetism of mesoscopic graphdiyne by doping with nitrogen
Source: Sci Rep. 2017 Sep 14;7:11535. doi: 10.1038/s41598-017-11698-9 (PMC5599548; doi:10.1038/s41598-017-11698-9)
Supplement: Supplementary file 1 — revised supporting information [file 41598_2017_11698_MOESM1_ESM.doc]

Enhanced paramagnetism of mesoscopic graphdiyne by doping with nitrogen

Mingjia Zhang,^1,+^ Xiaoxiong Wang,^2,+^ Huijuan Sun,^2^ Ning Wang,^1^ Qing Lv,^1^ Weiwei Cui,^1,2^ Yunze Long,^2^& Changshui Huang^1,*^

^1^Qingdao Institute of Bioenergy and Bioprocess Technology, Chinese Academy of Sciences, Qingdao 266101, P. R. China

^2^Department of Physics, Qingdao University, Qingdao 266071, P. R. China

*huangcs@qibebt.ac.cn

+these authors contributed equally to this work.


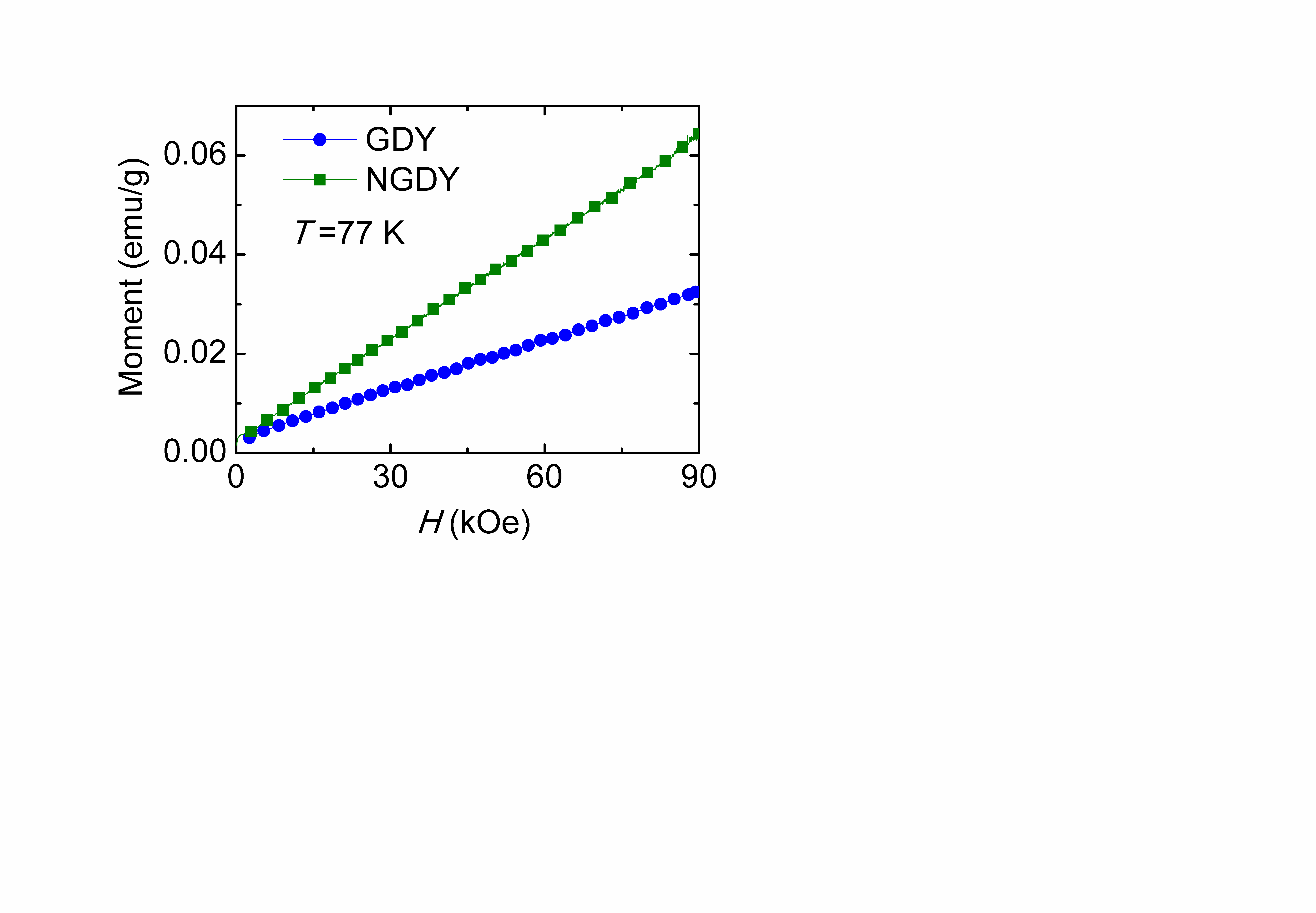


Figure S1 The M-H curves of GDY and N-GDY at liquid nitrogen temperature 77 K.


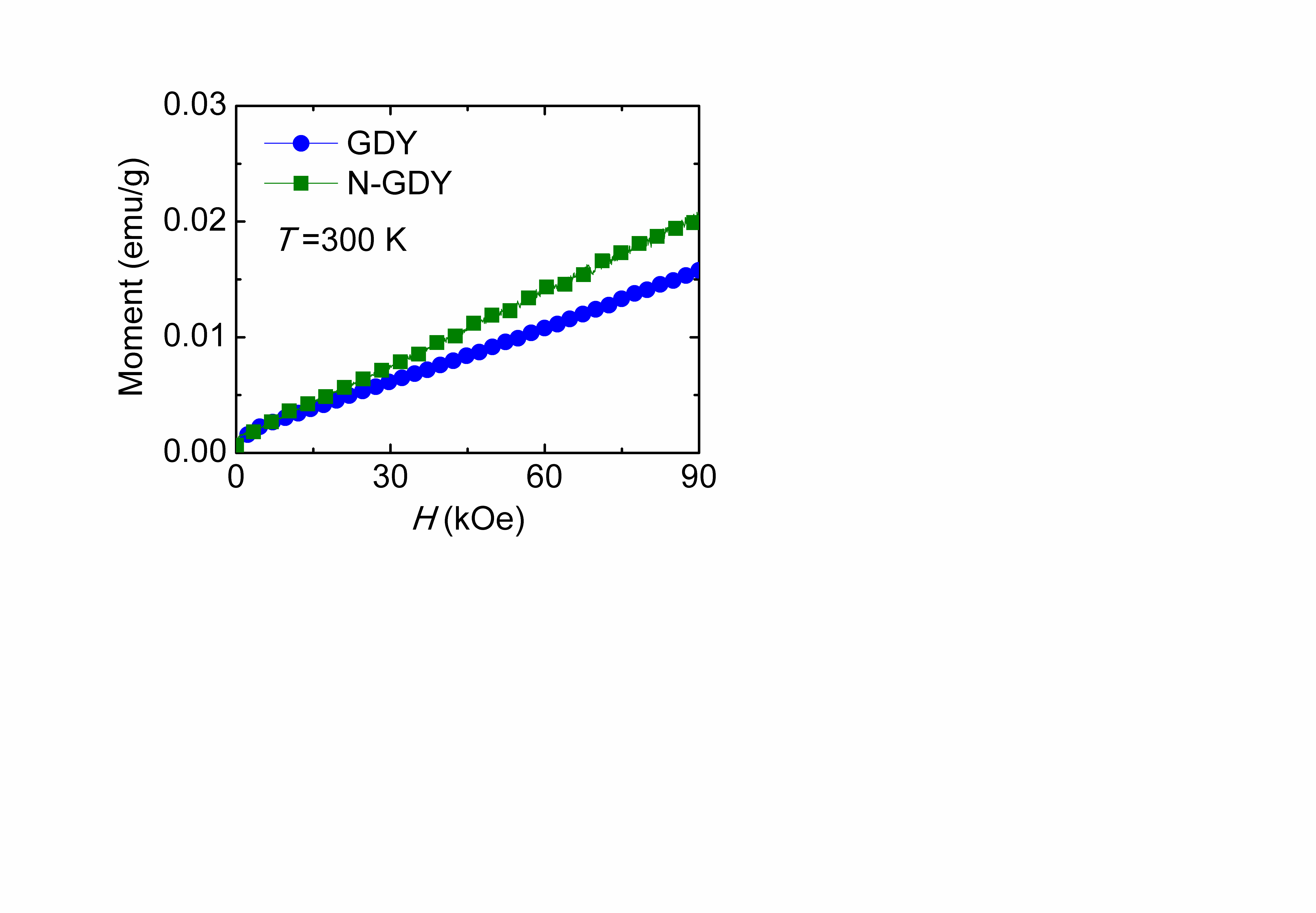


Figure S2 The M-H curves of GDY and N-GDY at room temperature 300 K.


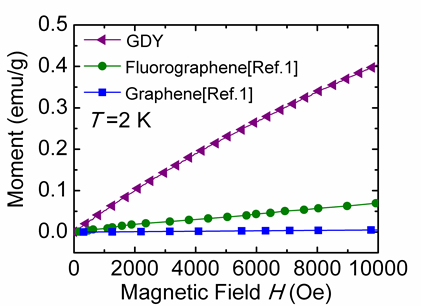


Figure S3 The M-H curves at 2 K of graphdiyne compared with that of graphene and fluorographene reported in Ref. 1.


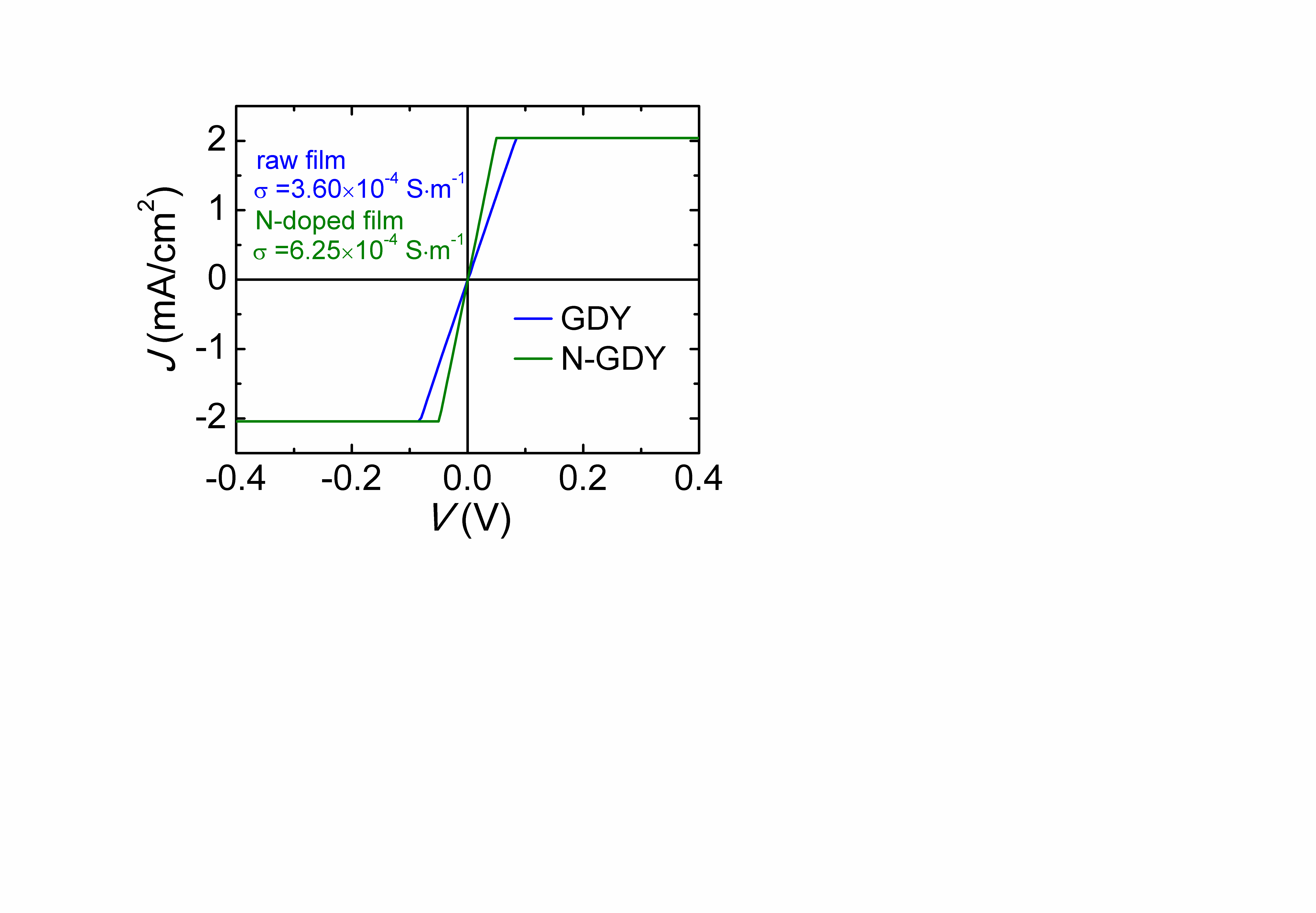


Figure S4 *J–V* curves of graphdiyne and N-doped graphdiyne films measurement on the devices respectively.


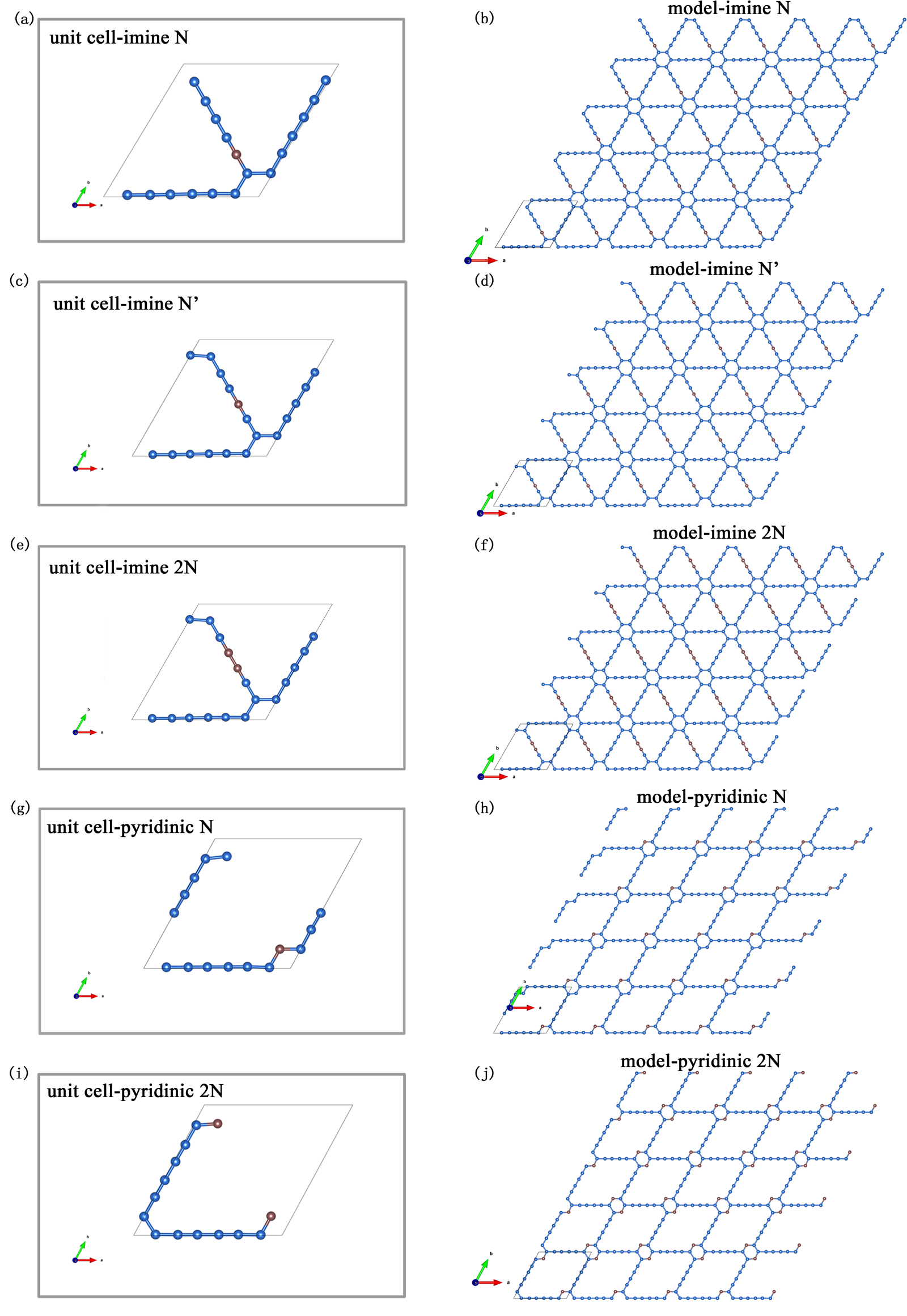


Figure S5 The models in DFT simulations for the typical configurations of N-doped graphdiyne respectively. Left displays the corresponding unit cell.


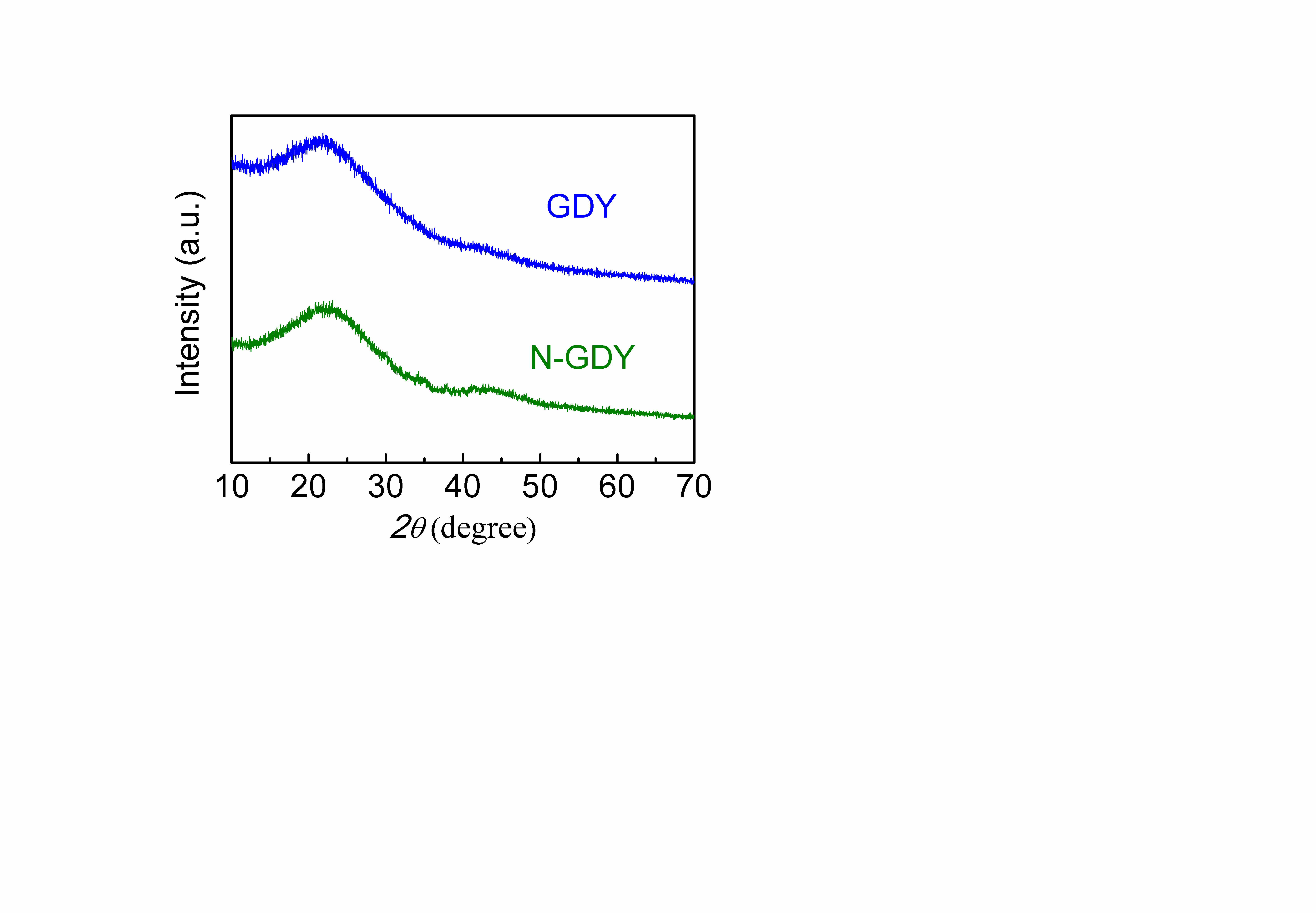


Figure S6 The XRD patterns of raw GDY powder and nitrogen doped GDY powder.

Reference

1. Q. Feng; N. J. Tang; F. C. Liu; Q. Q. Cao; W. H. Zheng; W. C. Ren; X. G. Wan; Du, Y. W., Obtaining High Localized Spin Magnetic Moments by Fluorination of Reduced Graphene Oxide. *ACS Nano* **2013**, *7*, 6729-6734.
